# Clinical effectiveness of an online supervised group physical and mental health rehabilitation programme for adults with post-covid-19 condition (REGAIN study): multicentre randomised controlled trial

**DOI:** 10.1136/bmj.q988

**Published:** 2024-05-01

Several sections of this paper by McGregor and colleagues (*BMJ* 2024;385:e076506, doi:10.1136/bmj-2023-076506, published 7 February 2024) have been updated for clarity of the study population. The conclusion in the abstract and the first and second points of the “What this study adds” subsection of the box should have made it clear that in adults with post-covid-19 condition “at least three months after hospital discharge for covid-19” the online, home based, supervised, group physical and mental health rehabilitation programme (REGAIN) showed clinical benefits and lack of harm.

